# Two new species of the genus *Caligus* (Crustacea, Copepoda, Siphonostomatoida) from the Sea of Japan, with a note on the establishment of a new species group

**DOI:** 10.3897/zookeys.893.46923

**Published:** 2019-12-02

**Authors:** Susumu Ohtsuka, Geoffrey A. Boxshall

**Affiliations:** 1 Graduate School of Integrated Sciences for Life, Hiroshima University, Kagamiyama 1-4-4, Higashi-Hiroshima, Hiroshima 739-8528, Japan Hiroshima University Hiroshima Japan; 2 Department of Life Sciences, Natural History Museum, Cromwell Road, London SW7 5BD, UK Natural History Museum London United Kingdom

**Keywords:** caligid copepods, plankton, *
Platycephalus
*, sea lice, taxonomy

## Abstract

Two new species of *Caligus* are described from the Japanese coast of the Sea of Japan. *Caligus chinglonglini***sp. nov.** is based on a male specimen found in a plankton sample, whereas *C. kajii***sp. nov.** was collected from the body surface of the host flathead *Platycephalus* sp. These two new species can be assigned to a distinct species group, the *pseudorhombi* group newly named and defined by the morphology of the genital complex in both sexes, and by the structure and armature of legs 2 and 4. The species group so far accommodates 19 species including these two new species. The morphology, host specificity and zoogeography of the species group are discussed herein and keys to species groups of *Caligus* and to species of the *C. pseudorhombi* species group are provided.

## Introduction

Members of the genus *Caligus* Müller, 1785 are known as sea lice and several species are known to cause serious economic losses in marine fish farming facilities (Ho and Lin 2004b; Johnson et al. 2004, Costello 2009; Shinn et al. 2015a, b). The life cycle of sea lice and their host specificity have been the subject of intensive studies designed to develop methods of controlling these pests. The life cycles of caligid copepods have been shown to be more diverse than expected. The general pattern is for a species to utilize a single host for all post-copepodid stages, after infection of the host fish (Ho and Lin 2004b; Dojiri and Ho 2013). A less common type of life cycle is found in the so-called “planktonic caligids” such as *Caligus undulatus* Shen & Li, 1959, *C. ogawai* Venmathi Maran, Ohtsuka & Shang, 2012 and *C. ilhoikimi* Suárez-Morales & Gasca, 2016, which exhibit a dual mode of life with adults found both on the host fish and free in the water column (Shen and Li 1959; Ho and Lin 2004a; Venmathi Maran and Ohtsuka 2008; Venmathi Maran et al. 2012a, b, 2016; Suárez-Morales et al. 2012a, b; Suárez-Morales and Gasca 2016; Kim et al. 2019). A third life cycle pattern is found in species which conduct host switching after the final molt of the chalimus phase and require both an intermediate and a final host (Hayward et al. 2009; Ohtsuka et al. 2018). The third type is rare and has so far been recorded only in three species infecting farmed fish (Hayward et al. 2008, Ohtsuka et al. 2018), although Cressey and Cressey (1980) suspected that the adults of *Caligus biseriodentatus* Shen, 1957 occurred on different host species from the immature stages.

An undescribed species of *Caligus* was found in a plankton sample collected at Ashibe Port, Iki Island, Nagasaki Prefecture, Japan on May 24, 2014. Another undescribed species was found infecting the flathead *Platycephalus* sp. caught off Shimonoseki City, Yamaguchi Prefecture, Japan in the Sea of Japan in 2016. Since these two undescribed species belong to a distinct species group of *Caligus*, they are described together in the present paper, together with remarks on taxonomy, host specificity and distribution of members of the species group.

## Materials and methods

A single male specimen of *Caligus* was found in a plankton sample collected by towing a small plankton net around an underwater fishing light (KU-5MB, Koto Electric Co., Ltd.) at Ashibe Port, Iki Island, Nagasaki Prefecture, Japan (33°48.54'N, 129°45.231'E) during the night-time of May 24, 2014. This becomes the holotype of a new species. A second undescribed species was found infecting the body surface of the flathead *Platycephalus* sp. (total length 58 cm) caught by fishing off Shimonoseki City, Yamaguchi Prefecture, Japan (34°00.686'N, 130°53.756'E) in the morning of August 24, 2016. The copepods were fixed in 70% ethanol immediately after capture. After immersing the copepod specimens in lactophenol, these were examined using Humes and Gooding’s (1964) slides on a differential interference microscope (BX-53, Olympus Co., Ltd.) equipped with a drawing tube. Body lengths were measured from the frontal margin of the cephalothorax to the posterior margin of the caudal ramus excluding the caudal setae. Protists epibiontic on the undescribed caligid collected from Iki Island were photographed with a digital camera (DP21, Olympus Co., Ltd.) attached to the microscope. Terminology essentially follows Ho and Lin (2004b).

Type specimens are deposited at the National Museum of Natural History and Science, Tsukuba, Japan (NSMT-Cr).

## Taxonomy

### Order Shiphonostomatoida Thorell, 1859

#### Family Caligidae Burmeister, 1835

**Genus *Caligus* Müller, 1785**

##### Caligus chinglonglini sp. nov.

Taxon classificationAnimaliaSiphonostomatoidaCaligidae

92683892-B77C-558D-A181-6F3E64493679

http://zoobank.org/41B191CB-B10D-4015-AC2D-B4407FDBE6D3

**Figure 1.**
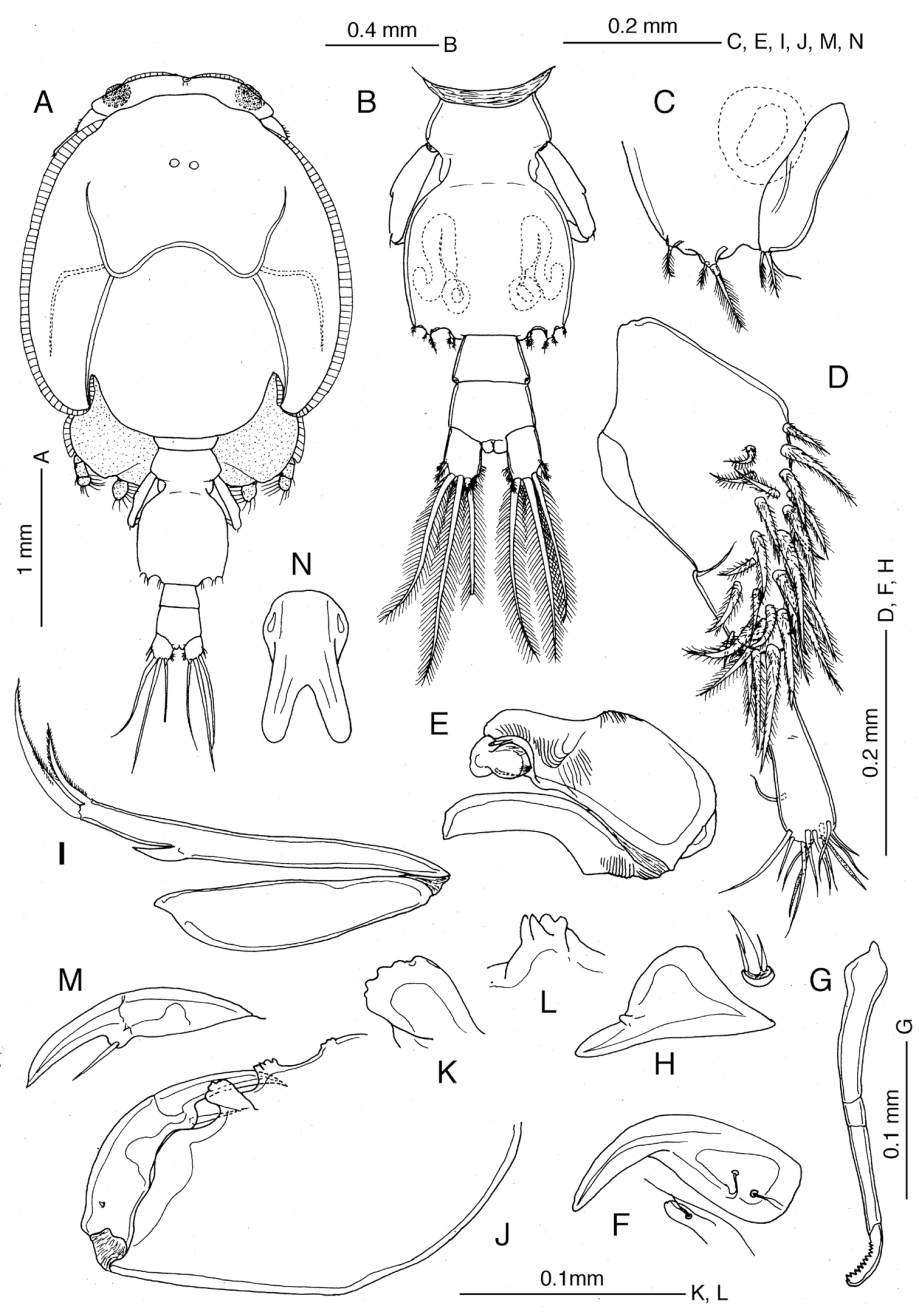
*Caligus chinglonglini* sp. nov., adult male, holotype **A** habitus, dorsal view **B** postcephalothoracic trunk, dorsal view **C** leg 5 and gonopore on right side, ventral view **D** antennule, ventral view **E** antenna **F** postantennal process **G** mandible **H** maxillule **I** maxilla **J** maxilliped **K** middle process on myxal margin of maxilliped corpus **L** distal process on myxal margin of maxilliped corpus **M** subchela of maxilliped **N** sternal furca.

**Figure 2.**
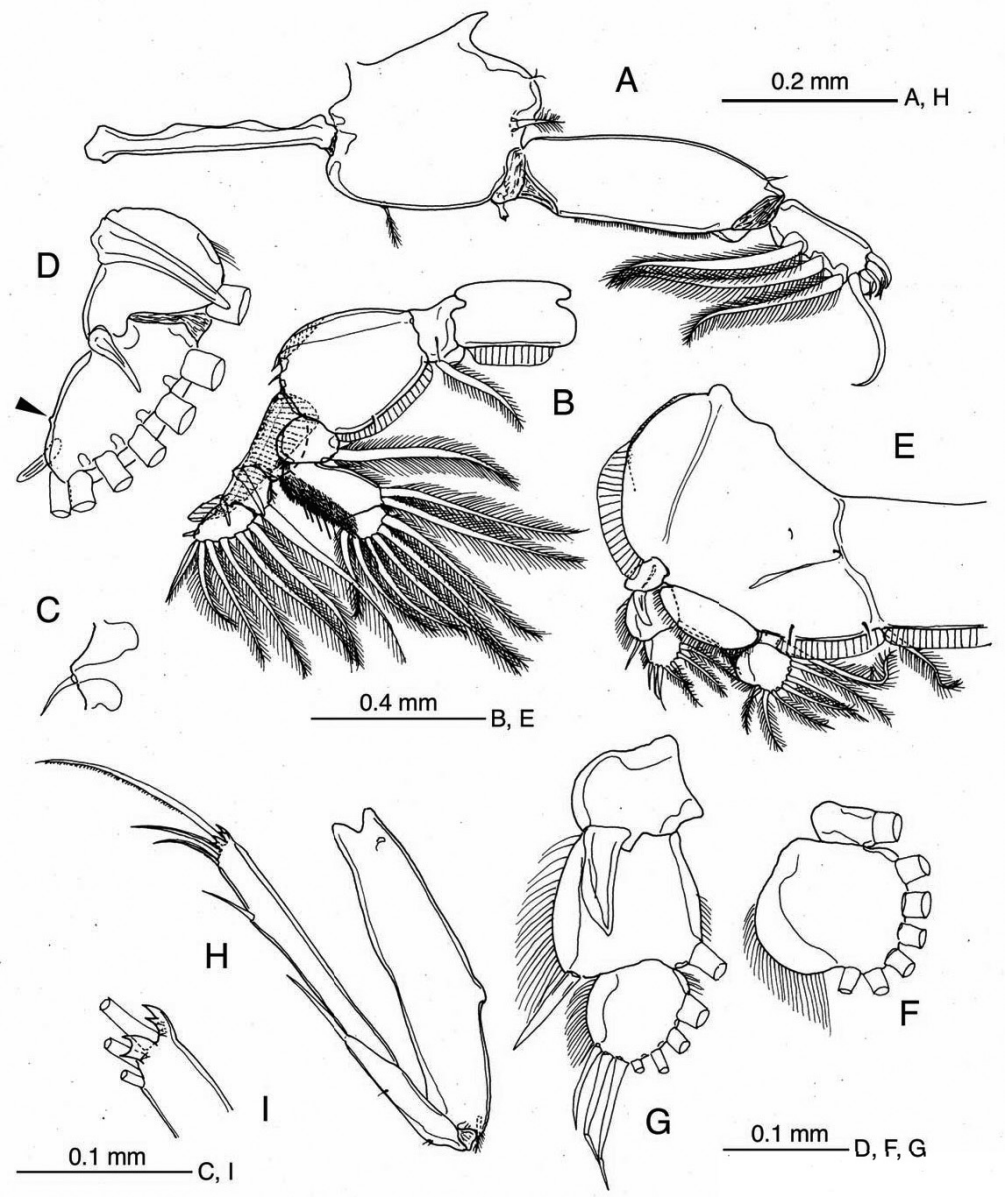
*Caligus chinglonglini* sp. nov., adult male, holotype **A** leg 1 **B** leg 2 **C** outer seta on basis of leg 2 **D** terminal part of exopod of leg 2, outer knob on segment 3 arrowed **E** leg 3 **F** endopod of leg 3 **G** exopod of leg 3 **H** leg 4 **I** terminal processes of leg 4.

**Figure 3.**
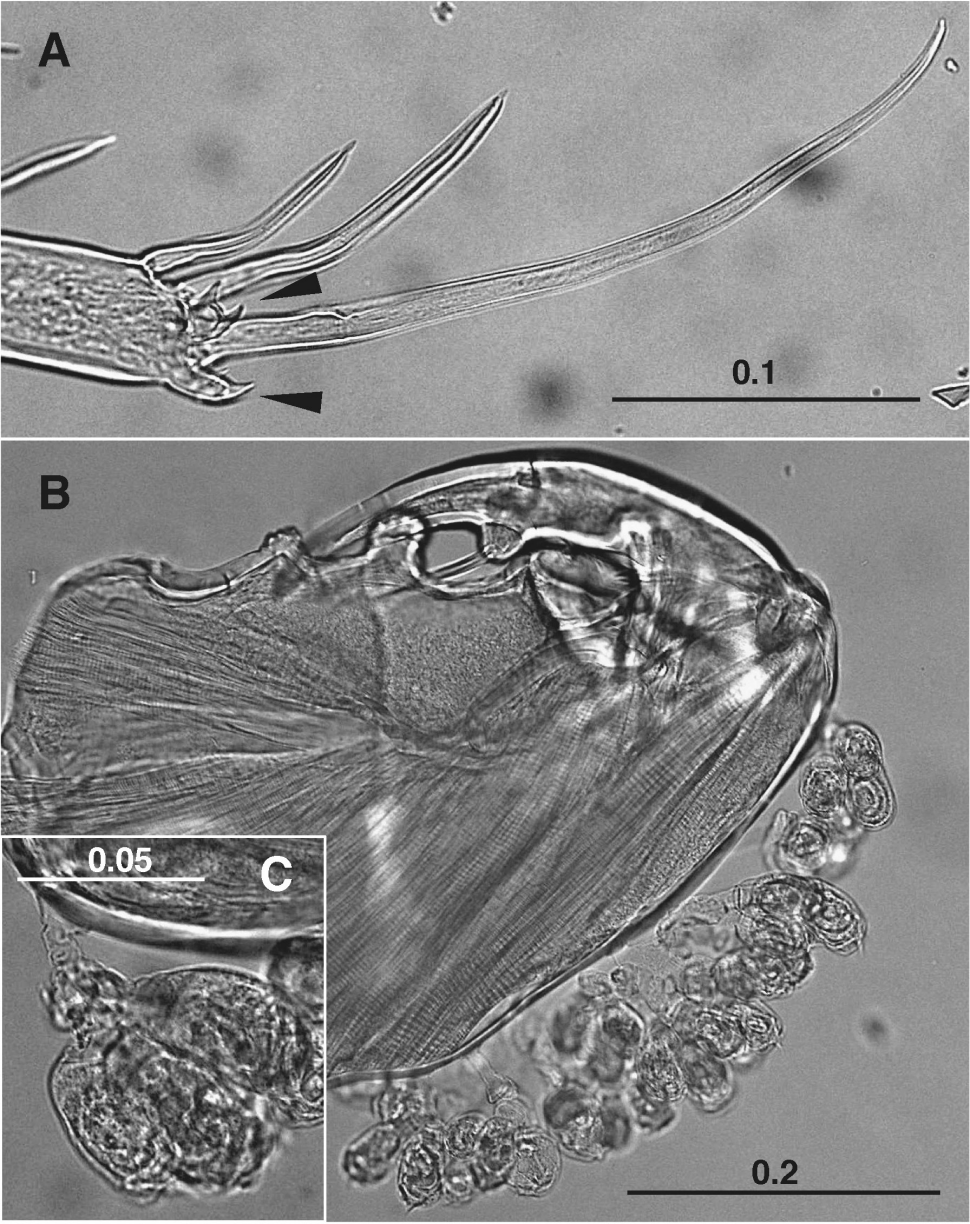
*Caligus chinglonglini* sp. nov., adult male, holotype **A** terminal part of leg 4, bifurcate processes arrowed **B** colony of peritrich ciliates attached along posterior margin of maxilliped corpus **C** peritrich ciliates attached on posterior margin of maxilliped corpus. Scale bars: in mm.

Figs 1 , 2 , 3 , Table 1

###### Material examined and type.

***Holotype*.** Japan • adult ♂; Ashibe Port, Iki Island, Nagasaki Prefecture (33°48.544'N, 129°45.231'E); night, May 24, 2014; partly dissected and mounted on 1 slide, body in vial (NSMT-Cr 26753); S. Ohtsuka leg.

###### Description.

**Male. *Body*** 4.02 mm long. Cephalothorax (Fig. 1A) slightly longer than wide. Pediger 4 (Fig. 1A, B) incompletely fused to genital complex. Genital complex (Fig. 1A, B) about 1.1 times wider than long, about 1.2 times longer than abdominal somites combined, produced posterolaterally into 2 knobs representing leg 5 (Fig. 1C), armed with 1 and 2 setae. Paired genital opercula (Fig. 1C) representing leg 6, each bearing 2 minute unequal setae terminally. Abdomen 2-segmented, second somite ca. 1.7 times longer than first. Caudal ramus (Fig. 1B) furnished with row of fine setules along inner margin; setae II and III located close together subterminally; setae IV–VI well developed; seta VII minute, located at inner distal corner.

Antennule (Fig. 1D) 2-segmented; proximal segment with 26 setae, distal segment with 11 setae and 2 aesthetascs. Antenna (Fig. 1E) 3-segmented; proximal segment unarmed, with adhesion pad distally; middle segment massive, unarmed, with 2 adhesion pads at mid-length and 1 pad terminally; distal segment small, multi-layered flap with 2 small setae proximally. Postantennal process (Fig. 1F) moderately curved, with 2 bisensillate papillae proximally plus bisensillate papilla on adjacent ventral cephalothoracic surface. Mandible (Fig. 1G) with 11 teeth on margin subterminally. Maxillule (Fig. 1H) represented by anterior papilla with 1 thick and 2 fine setae and posterior dentiform process with rounded prominence subterminally. Maxilla (Fig. 1I) 2-segmented, lacertus (syncoxa) unarmed, brachium (basis) slender, with large hyaline membrane at terminal third, plus long calamus and short cana apically. Maxilliped (Figs 1J–M, 3B) heavily chitinized, 2-segmented; corpus (protopod) massive, with 3 unequal processes along myxal margin, proximal process low, middle process with multiple tips, distal process largest, with irregular, undulating distal margin; shaft (endopod) as long as and incompletely fused to claw to form subchela; barbel located on rounded inner basal process of claw. Sternal furca (Fig. 1N) with divergent tines originating close together, rounded at tip.

Armature and elements of legs 1–4 as in Table 1. ***Leg 1*** (Fig. 2A) with massive protopod bearing 1 inner and 1 outer small plumose setae plus bifid setule on outer margin; intercoxal sclerite slender, unornamented; endopod reduced to club-shaped process located near base of exopod; exopod 2-segmented, first segment with row of fine setules along inner margin and 1 naked seta at outer distal corner, second segment with 3 large plumose setae along inner (posterior) margin and 4 elements terminally, middle two of which each bearing accessory process. ***Leg 2*** (Fig. 2B–D) with intercoxal sclerite ornamented with trapezoidal marginal membrane along posterior margin; coxa with large plumose seta at posterior corner and minute setule on anterior surface; basis ornamented with marginal membrane on both inner and outer edges, bearing 1 minute seta on knob at outer distal corner (Fig. 2C) plus setule near midpoint of inner margin; endopod 3-segmented, outer margins of second and third segments with dense patches of minute setules; exopod 3-segmented, first segment with long outer spine directed obliquely across surface of second segment, second segment with relatively short outer spine, third segment with small outer knob (arrowed in Fig. 2D), 1 reduced outer spine, 1 short terminal spine and 5 plumose setae increasing in size from apical to innermost. ***Leg 3*** (Fig. 2E–G) apron (protopod) without surface processes, bearing well developed inner seta and 1 minute outer seta, plus 2 relatively long setules along posterior margin; outer basal margin of apron undulating; endopod 2-segmented, proximal segment small, with 1 long plumose seta; velum developed, hirsute along free posterior margin; second segment expanded along outer margin (Fig. 2F); exopod 3-segmented (Fig. 2G), proximal segment small, with slightly curved outer spine not reaching distal border of next segment, middle segment with 1 inner plumose and 1 outer naked seta, third segment with 3 spiniform setae increasing in size distally plus 4 inner setae.

***Leg 4*** (Figs 2H, I, 3A) with protopod bearing low outer prominence at mid-length and minute plumose seta at outer distal corner; exopod distinctly 2-segmented, first exopodal segment with long outer spine almost fused basally to segment and reaching more than half distance to origin of proximalmost outer spine on compound second segment; second segment with 1 terminal and 2 slender spines on distal margin plus lateral spine, plus 2 bifurcate processes terminally; each process complex, with 1 or 3 minute prominences basally (Fig. 2I).

***Leg 5*** (Fig. 1C) represented by 2 small knobs, outer knob bearing protopodal seta, inner knob representing exopod, bearing 2 plumose setae terminally. Leg 6 (Fig. 1C) consisting of genital operculum, bearing 2 terminal minute setae.

**Female.** Unknown.

###### Remarks.

The new species is most closely related to *C. acanthopagri* Ho, Lin & Chen, 1994, *C. dieuzeidei* sensu Shiino (1954b), and *C. latigenitalis* Shiino, 1954 in general appearance and in the structure of the appendages and sternal furca. As Izawa and Choi (2000) and Ho and Lin (2004b) suggested, the minor but most distinct difference can be found in the structure of the pectens of the second exopod segment of leg 4 among these three species. Those of *C. chinglonglini* sp. nov., *C. dieuzeidei* sensu Shiino (1954b) (see Discussion), and *C. latigenitalis* are sharply indented, whereas that of *C. acanthopagri* is composed of a hyaline membrane. The former three species can be distinguished by the number and shape of dentate processes (divided into 3 or 5 prominences but not hand-like in *C. chinglonglini*; 3 or 4 and hand-like in *C. dieuzeidei* sensu Shiino; 4 or 5 and hand-like in *C. latigenitalis*). In addition, the shape and numbers of processes of the maxillipedal myxal area differ among the males of these three species. In *C. chinglonglini* sp. nov. and *C. latigenitalis*, there are 3 processes arrayed along the myxal margin, but the middle process is furnished with serrated tips in the former but is rounded in the latter. In *C. dieuzeidei* sensu Shiino (1954b), there are only two processes, one quadrangular and the other low triangular, present along the margin.

Although the present new species is described on the basis of a single male, no other species belonging to the newly proposed *pseudorhombi* species group (see Discussion) has so far been recorded from Japanese waters except for *C. latigenitalis* (Nagasawa et al. 2010) in which only females were originally described by Shiino (1954a) and subsequently both sexes were redescribed in detail by Izawa and Choi (2000). These two species are distinguishable as mentioned above. In addition, *C. bifurcus* Shen, 1958, assigned to the same species group was described from Chinese waters based only on two females, but the non-sexually dimorphic characteristics such as sternal furca and legs differ distinctly from those of *C. chinglonglini* sp. nov. Therefore, the establishment of the present new species is justified.

The new species is the fourth species of *Caligus* found exclusively from plankton samples in Japan (see Venmathi Maran et al. 2016, table 2).

Peritrich ciliates were attached along the posterior margin of both maxillipeds (Fig. 3B, C) and on the ventral side of the cephalothorax. Epibiont suctorian and peritrich ciliates have already been recorded from species of *Caligus* and *Lepeophtheirus* von Nordmann, 1832 (Stone and Bruno 1989; Gresty and Warren 1993; Fernandez-Leborans et al. 2005). In *L. salmonis* (Krøyer, 1837) collected from Hokkaido, Japan, the peritrich *Epistylis* sp. attached mainly to the antennae and legs 2 and 3. This is the first record of the occurrence of epibiont peritrich ciliates on “pelagic caligids” (Ho and Lin 2004a; Venmathi Maran and Ohtsuka 2008; Venmathi Maran et al. 2012a, b, 2016).

**Table 1.** Armature and elements of legs 1 to 4 of *Caligus chinglonglini* sp. nov.

	Protopod (coxa; basis)	Endopod	Exopod
Leg 1	1-1	(vestigial)	1-0; III, 1, 3
Leg 2	0-1; 1-0	0-1; 0-2; 6	I-1; I-1; I, I,5
Leg 3	1-1	0-1; 6	I-0; I-1; 3, 4
Leg 4	1-0	(absent)	1-0; I, III

###### Etymology.

The new species is named in honor of the late Dr Ching-long Lin who made a great contribution to the taxonomy of parasitic copepods together with Prof. Ju-shey Ho.

##### Caligus kajii sp. nov.

Taxon classificationAnimaliaSiphonostomatoidaCaligidae

1490FC06-A592-512B-8B14-F71B4E47B420

http://zoobank.org/9A3A7B46-6140-4865-AAFA-99E718FBA8D6

**Figure 4.**
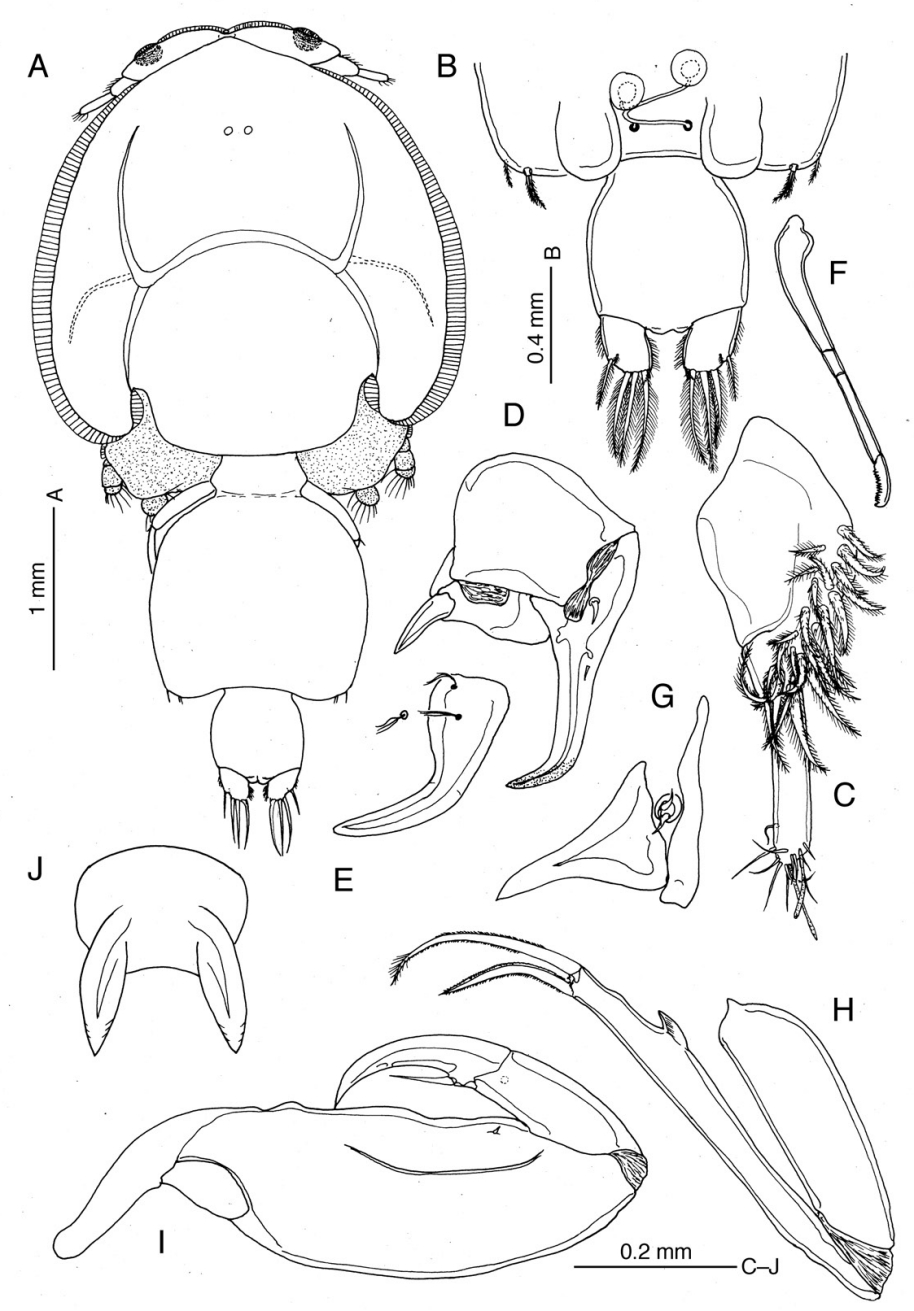
*Caligus kajii* sp. nov., adult female, paratype **A** habitus, dorsal view **B** posterior end of genital complex plus abdomen, ventral view **C** antennule **D** antenna **E** postantennal process **F** mandible **G** maxillule **H** maxilla **I** maxilliped **J** sternal furca.

**Figure 5.**
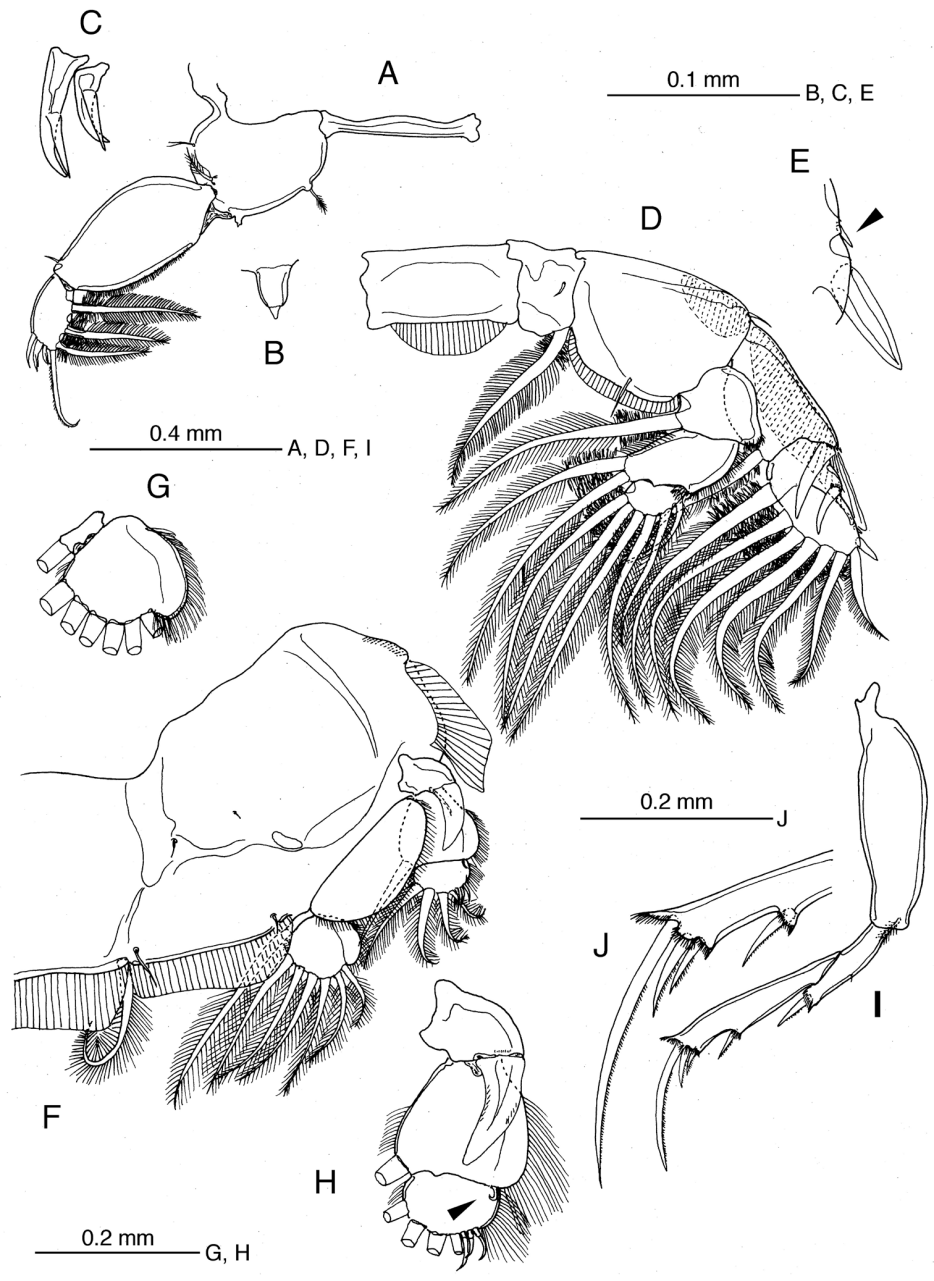
*Caligus kajii* sp. nov., adult female, paratype **A** leg 1 **B** endopod of leg 1 **C** two terminal spines of second exopod segment of leg 1 **D** leg 2 **E** outer spines of terminal exopod segment of leg 2, reduced spine arrowed **F** leg 3 **G** endopod of leg 3 **H** exopod of leg 3 **I** leg 4 **J** terminal part of leg 4.

**Figure 6.**
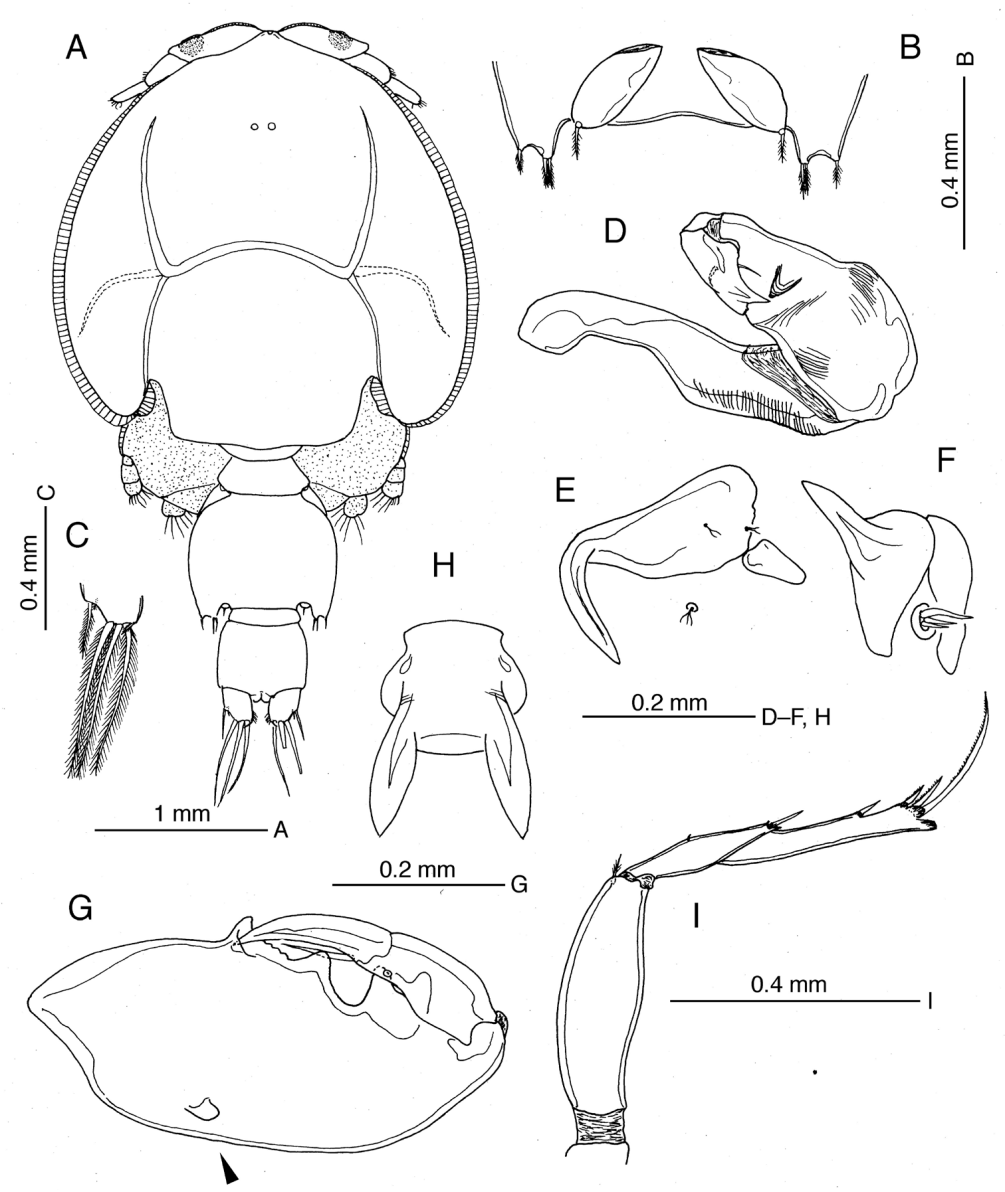
*Caligus kajii* sp. nov., adult male, allotype **A** habitus, dorsal view **B** gonopores, ventral view **C** tip of caudal ramus, dorsal view **D** antenna **E** postantennal process **F** maxillule **G** maxilliped, rounded knob on posterior surface of corpus arrowed **H** sternal furca **I** leg 4.

Figs 4 , 5 , 6 , Table 2

###### Material examined.

JAPAN • 38 adult ♀♀ and 14 adult ♂♂; parasitic on body surface of *Platycephalus* sp. (total length 58 cm) collected from a depth of 15 m off Shimonoseki, Yamaguchi Prefecture (34°00.686'N, 130°53.756'E); morning of August 24, 2016; S. Ohtsuka leg.

###### Types.

***Holotype*.** JAPAN •1 ovigerous adult ♀; parasitic on body surface of *Platycephalus* sp. (total length 58 cm) collected from a depth of 15 m off Shimonoseki, Yamaguchi Prefecture (34°00.686'N, 130°53.756'E); morning of August 24, 2016; whole specimen (NSMT-Cr 26754); S. Ohtsuka leg. ***Allotype*.** JAPAN•1 adult ♂, same data as in holotype; partly dissected on 1 slide, body in vial (NSMT-Cr 26755); S. Ohtsuka leg. ***Paratypes*.** JAPAN•1♀, same data as in holotype; partly dissected and bodies in vials (NSMT-Cr 26756); 36♀♀ and 13♂♂, same data as in holotype; whole specimens (NSMT-Cr 26757); S. Ohtsuka leg.

###### Description.

**Female.** Body length of holotype 6.16 mm, 4.86–6.16 mm in holotype and female paratypes (mean ± standard deviation = 5.49 ± 0.32 mm, *N* = 38). Dorsal cephalothoracic shield subcircular, almost as long as wide (Fig. 4A). Lunules (Fig. 4A) relatively small. Pediger 4 almost completely fused to genital complex. Genital complex (Fig. 4A) subquadrate, about 1.14 times longer than wide, produced posteroventrally into pair of rounded processes between which paired copulatory pores located (Fig. 4B). Spermatophores (Fig. 4B) attached to copulatory pores via fine tubules; spermatophore proper globular, ca. 0.12 mm in diameter. Paired egg strings of holotype female containing 22 and 24 eggs. Abdomen (Fig. 4A, B) 1-segmented, about as long as wide. Caudal ramus (Fig. 4A, B) furnished with rows of setules along inner and outer margins; seta II minute, located near base of seta III on subterminal ventral surface, seta III subterminal, setae IV-VI terminal, well developed, seta VII minute, located at inner distal corner.

Antennule (Fig. 4C) 2-segmented; proximal segment bearing 26 setae on anteroventral surface; distal segment with 1 subterminal and 11 terminal setae and 2 short aesthetascs. Antenna (Fig. 4D) 3-segmented, heavily sclerotized; proximal segment with acutely pointed posterior process; middle segment subrectangular, unarmed; distal segment abruptly curved inward at distal quarter, armed with proximal seta and minute middle seta. Postantennal process (Fig. 4E) as long as distal segment of antenna, smoothly curved inward, with 2 multisensillate papillae basally; similar multisensillate papilla located adjacent to base of process. Mandible (Fig. 4F) with distal part bearing 12 teeth. Maxillule (Fig. 4G) consisting of anterior papilla bearing 3 setae of unequal length and triangular, plate-like posterior process. Maxilla (Fig. 4H) 2-segmented, lacertus (syncoxa) unarmed; brachium (basis) ca. 1.5 times longer than lacertus, with flabellum at about anterior one-third of length; calamus about 1.5 times longer than canna. Maxilliped (Fig. 4I) heavily sclerotized; corpus (protopod) elongate, about 1.7 times as long as subchela, with low proximal lobe located at about 30% along myxal margin, plus minute setule in distal quarter of corpus; shaft (endopod) and claw incompletely fused; claw with antero-proximal barb reaching beyond mid-length. Sternal furca (Fig. 4J) with tines widely separated at base and bluntly pointed.

Armature and elements of legs 1–4 as in Table 2. ***Leg 1*** (Fig. 5A–C) with protopod bearing 1 marginal bifurcate setule and 2 surface setae; endopod reduced to small knob with vestigial element at tip (Fig. 5B); exopod 2-segmented, proximal segment with row of setules along inner margin and outer distal seta, distal segment with 3 plumose setae along inner margin and 1 naked outer spine, 2 terminal spines each with accessory process (Fig. 5C), plus long spinulose seta (seta 4) terminally. ***Leg 2*** (Fig. 5D, E) with intercoxal sclerite bearing semi-circular marginal membrane along posterior margin; coxa with large plumose seta at posterior corner and setule on anterior surface; basis ornamented with marginal membrane along both inner and outer edges and long setule near midpoint of inner margin; armed with minute seta at distal outer corner; rami 3-segmented; first endopod segment with notch bearing tuft of setules, second segment furnished with row of setules along outer margin, third segment with tuft of setules near base of proximal outer seta; first exopod segment with anterior marginal membrane reflexed dorsally over segment surface and long, stout outer spine directed obliquely across surface of second segment; second segment with smaller outer spine than in first segment; third segment armed with 1 reduced spine (arrowed in Fig. 5E) and 1 moderate-size outer spine. ***Leg 3*** (Fig. 5F–H) apron (protopod) with no distinct ornamentation on surface, armed with 1 long inner seta and 1 small outer seta terminally; endopod 2-segmented, proximal segment reduced, velum developed, decorated entirely with row of setules along free posterior margin; distal segment with outer margin expanded and hirsute Fig. 5G; exopod 3-segmented, first segment with strong, slightly curved, inward-directed outer spine not reaching distal margin of second segment, second segment with expanded, hirsute outer margin and 1 minute outer seta (arrowed in Fig. 5H), third segment with 3 small spines plus 4 inner setae.

***Leg 4*** (Fig. 5I, J) protopod slightly shorter than exopod, bearing minute plumose seta at outer distal corner; exopod 2-segmented, with pecten at base of each exopodal spine; first exopodal segment bearing spinulose spine reaching nearly half of distance to proximalmost outer spine on second segment; innermost terminal spine more than 3 times longer than middle spine. Leg 5 (Fig. 4B) represented by small outer knob bearing protopodal seta and inner (exopodal) knob bearing 2 setae.

**Male.** Body length of allotype 4.36 mm, range 4.09–5.73 mm long in allotype plus all male paratypes (4.69 ± 0.51 mm long, *N* = 14). Cephalothorax (Fig. 6A) as in female. Pediger 4 (Fig. 6A) separate from genital complex, wider than long. Genital complex (Fig. 6A, B) about 1.4 times wider than long and about 1.2 times longer than abdominal somites combined, expanded posterolaterally into 2 knobs (leg 5), outer knob bearing 1 seta, inner (exopodal) knob with 2 setae; paired genital opercula representing leg 6 (Fig. 6B), each bearing 1 small terminal seta. Abdomen (Fig. 6A) 2-segmented, first segment small, second segment ca. 4.8 times longer than first. Caudal ramus (Fig. 6C) as in female.

Antennule, mandible, maxillule (Fig. 6F) and maxilla as in female. Antenna (Fig. 6D) 3-segmented; proximal segment long, unarmed, with corrugated pad distally; middle segment massive, with 1 proximal, 2 middle and 2 subterminal corrugated pads; distal segment short, with 2 fine setae at mid-length, 1 bluntly pointed process terminally and 1 membranous truncate projection subterminally. Postantennal process (Fig. 6E) similar to that of female, but more abruptly curved inward than in female. Maxilliped (Fig. 6G) stouter than in female; corpus with rounded knob proximally on posterior surface (arrowhead in Fig. 6G) and 3 distinct processes in myxal area, proximalmost process socket-like, to receive distal tip of subchela; shaft and claw partly fused, ca. 0.67 times as long as corpus; barb about half length of claw.

**Table 2.** Armature and elements of legs 1 to 4 of *Caligus kajii* sp. nov.

	Protopod (coxa; basis)	Endopod	Exopod
Leg 1	1-1	(vestigial)	1-0; III, 1, 3
Leg 2	0-1; 1-0	0-1; 0-2; 6	I-1; I-1; II, I,5
Leg 3	1-1	0-1; 6	I-0; 1(vestigial)-1; III, 4
Leg 4	1-0	(absent)	1-0; I, III

###### Remarks.

The female of the new species most closely resembles *Caligus bifurcus*, *C. musaicus* Cavaleiro, Santos & Ho, 2010, *C. pectinatus* Shiino, 1965, *C. pseudorhombi* Boxshall, 2018, *C. pterois* Kurian, 1949 and *C. xystercus* Cressey, 1991. All these species share a 2-segmented exopod on leg 4 armed with 4 spines on the distal exopodal segment, the female genital complex is nearly as long as wide and about twice as long as the abdomen, and the abdomen is about as long as wide. However, the present new species is distinguished from these species by the combination of the following characteristics: (1) the genital complex is as long as wide (cf. wider than long in *C. bifurcus*, *C. musaicus* and *C. pterois*; slightly longer than wide in *C. xystercus*); (2) the genital complex is about 2.1 times longer than the abdomen (cf. 1.2 times longer in *C. bifurcus*; 2.2 in *C. musaicus*; 2.1 in *C. pseudorhombi*; 1.6 in *C. pterois*; 3.6 in *C. xystercus*); (3) the corpus of the maxilliped lacks processes (cf. ridge-like process present in *C. pseudorhombi* and *C. pterois*); (4) the tines of the sternal furca taper distally (cf. uniform in width and with a truncate tip in *C. pectinatus*); (5) the terminal exopod segment of leg 1 is furnished with 3 large spines terminally (2 in *C. pseudorhombi*); and (6) the maxillipedal subchela is more than half the length of the corpus (cf. much shorter in *C. musaicus*).

In males, the new species is most similar to *C. musaicus*, *C. nuenonnae* Andrews, Bott, Battaglene & Nowak, 2009, *C. pterois*, and *C. priacanthi* Pillai, 1962. These five species share the following characteristics: (1) the genital complex is laterally expanded and produced into 2 posterolateral protuberances representing leg 5, armed with 1 (outer lobe) and 2 setae (inner lobe); (2) the abdomen is completely or incompletely 2-segmented and shorter than the genital complex; (3) the maxillipedal corpus is well developed and carries anteriorly-produced processes on the myxal surface. However, the new species is easily distinguishable from these congeners by the combination of the following features: (1) the maxillipedal corpus has a rounded process on the posterior surface (absent in the other species); (2) the sternal furca has pointed tines which are widely separated (tines that are close at base and with rounded tips in *C. priacanthi*; rounded in *C. nuenonnae*); (3) the mxyal surface of the maxilliped carries 3 large, rounded processes along the margin, (cf. the processes are different in shape and number in the other species); (4) the posterior dentiform process of the maxillule lacks a surface ornamentation of minute prominences (present in *C. nuenonnae*).

###### Etymology.

The new species is named in honor of the late, supremely talented carcinologist Tomonari Kaji who passed away in May 2019.

#### Key to species groups currently recognized within the genus *Caligus*

**Table:** 

1	Leg 1 with 3 inner setae on distal exopod segment lost or highly reduced	***C. productus* group**
–	Leg 1 with 3 inner setae on distal exopod segment well developed	**2**
2	Large denticles present along outer margin of second endopod segment of leg 2	***C. bonito* group**
–	Large denticles absent along outer margin of second endopod segment of leg 2	**3**
3	Leg 4 is 4-segmented (3-segmented exopod)	**4**
–	Leg 4 is 3-segmented (2-segmented exopod)	**5**
4	Long-bodies; apron of leg 3 with raised bifid cuticular rib and rosette-like array of denticles	***C. confusus* group**
–	Compact bodies; apron of leg 3 without such ornamentation	***C. diaphanus* group**
5	Distal exopod segment of leg 4 with 1 outer and 3 terminal spines	***C. pseudorhombi* group**
–	Distal exopod segment of leg 4 with 3 terminal spines	***C. macarovi* group**

#### Key to species within the *C. pseudorhombi* group

**Female**

**Table:** 

1	Tines of sternal furca widely separated	**2**
–	Tines of sternal furca originating close together	**9**
2	Outermost spine of terminal exopod segment of leg 1 reduced or absent	**3**
–	Outermost spine of terminal exopod segment of leg 1 developed	**4**
3	Outermost spine of terminal exopod segment of leg 1 reduced	***C. pseudorhombi***
–	Outermost spine of terminal exopod segment of leg 1 absent	***C. xystercus***
4	Maxillipedal corpus with triangular process at mid-length	***C. pterois***
	Maxillipedal corpus without distinct process	**5**
5	Tines of sternal furca with truncate tips	**6**
–	Tines of sternal furca with pointed tips	**7**
6	First segment of antenna with sharply pointed posterior process	***C. pectinatus***
–	First segment of antenna with bluntly pointed posterior process	***C. similis***
–	First segment of antenna with spatulate posterior process	***C. nuenonnae***
7	Tines of sternal furca swollen at mid-length	***C. kajii* sp. nov.**
–	Tines of sternal furca tapering distally	**8**
8	Gap between tines narrower than length of tine	***C. bifurcus***
–	Gap between tines wider than length of tine	***C. musaicus***
9	Maxillipedal corpus with conical process midway	**10**
–	Maxillipedal corpus without distinct process	**11**
10	Genital complex as long as wide	***C. dieuzeidei***
–	Genital complex wider than long	***C. priacanthi***
11	Postantennal process with accessory process basally	***C. hobsoni***
–	Postantennal process without accessory process	**12**
12	Genital complex more than twice as long as abdomen	**13**
–	Genital complex about 1.4 times longer than abdomen	***C. ligatus***
–	Genital complex about 1.7 times longer than abdomen	***C. longirostris***
13	First segment of antenna with spatulate posterior process (spine)	***C. buechlerae***
–	First segment of antenna with pointed posterior process (spine)	**14**
14	Tines of sternal furca short, thick, about 1.6 times as long as wide	***C. latigenitalis***
–	Tines of sternal furca long, slender, about 2.7 times as long as wide	***C. olsoni***

**Male**

**Table:** 

1	Both anterior and posterior knobs representing leg 5 produced posteriorly; leg 6 (genital operculum) with 1 (or rarely 2) small setae terminally	**2**
–	Only posterior knob representing leg 5 distally produced; leg 6 with 2 (or rarely 3) setae terminally	**7**
2	Tines of sternal furca widely separated	**3**
–	Tines of sternal furca close together	***C. priacanthi***
3	Posterior dentiform process of maxillule covered with minute prominences on medial and apical surfaces	***C. nuenonnae***
–	Posterior dentiform process of maxillule lacking such prominences	**4**
4	Maxilliped with rounded process proximally on posterior surface of corpus	***C. kajii* sp. nov.**
–	Maxilliped lacking such process on posterior surface of corpus	**5**
5	Third segment of antenna with long hook-like process terminally	***C. pterois***
–	Third segment of antenna with short claw	**6**
6	Maxillipedal corpus with 1 small dentiform and 1 bipartite process in myxal area	***C. musaicus***
–	Maxillipedal corpus with 2 spinous processes proximally and 1 rounded process distally along myxal margin	***C. pseudorhombi***
7	Genital complex almost as long as abdomen	**8**
–	Genital complex longer than abdomen	**12**
8	Tines of sternal furca slender with rounded tip	**10**
–	Tines of sternal furca thick with truncate or spatulate tip	**11**
10	Maxillipedal corpus with distinct process at mid-level	***C. ligatus***
–	Maxillipedal corpus without process	***C. longirostris***
11	Tines of sternal furca with truncate tip	***C. similis***
–	Tines of sternal furca with spatulate tip	***C. hobsoni***
12	Caudal ramus about 2 times longer than wide	***C. dieuzeidei***
–	Caudal ramus almost as long as wide	**13**
13	Maxillipedal corpus with inner process at mid-length	***C. olsoni***
–	Maxillipedal corpus with 3 inner processes along myxal margin	**14**
14	Terminal exopod segment of leg 4 with dentate processes at base of each terminal spine	**15**
–	Terminal exopod segment of leg 4 with hyaline membrane at base of each terminal spine	***C. acanthopagri***
15	Four or five tips on dentate processes at base of terminal spines of terminal exopod segment of leg 4	***C. latigenitalis***
–	Two tips on dentate processes at base of terminal spines of terminal exopod segment of leg 4	***C. chinglonglini* sp. nov.**

## Discussion

Five named species groups were recognized within the genus *Caligus* by Boxshall (2018) but as part of the justification for establishing *C. pseudorhombi* Boxshall, 2018 as a new species, he informally recognized an additional distinct species group. This unnamed species group was diagnosed on the basis of female morphology. The following features are shared by species within the group: (1) the exopod of leg 4 is 2-segmented and the compound distal segment carries 4 spines; (2) the genital complex of the female is as long as wide, without posterolateral lobes, and about twice as long as the abdomen; and (3) the abdomen is about as long as wide (see Boxshall 2018). An additional characteristic which we identify here relates to leg 2: the proximal spine on the outer margin of the third exopodal segment is markedly reduced and the adjacent distal spine is also relatively small in almost all members of the species group for which information on leg 2 is available. The males of the species group are defined for the first time as follows: (1) leg 4 is as in the female; (2) the genital complex is subquadrate with legs 5 and 6 located close together at the posterolateral corner; (3) the abdomen is 1- or, typically, 2-segmented; and (4) the myxal surface of the maxilliped has 1 to 3 pointed or rounded processes (except for *C. longirostris* Hewitt, 1964).

We recognize that the following 19 species can be included in this species group: *C. acanthopagri* (♀♂ known), *C. bifurcus* (♀), *C. buechlerae* Hewitt, 1964 (♀♂), *C. chinglonglini* sp. nov. (♂), *C. dieuzeidei* Brian, 1932 (♀♂), *C. hobsoni* Cressey, 1969, (♀♂), *C. kajii* sp. nov. (♀♂), *C. latigenitalis* (♀♂), *C. ligatus* (♀♂), *C. longirostris* (♀♂), *C. musaicus* (♀♂), *C. nuenonnae* (♀♂), *C. olsoni* Pearse, 1953 (♀♂), *C. pectinatus* (♀), *C. pseudorhombi* (♀♂), *C. priacanthi* (♀♂), *C. pterois* (♀♂), *C. similis* Ho, Kim & Nagasawa, 2005 (♀♂), and *C. xystercus* (♀). Unfortunately for *C. olsoni*, no information is available on leg 2. This species group is newly named as the *pseudorhombi* group, partly because it was first pointed out when *C. pseudorhombi* was originally described by Boxshall (2018), and partly because both sexes of the species were described in detail by Boxshall (2018).

Both sexes of *C. dieuzeidei* were described by Brian (1932) based on material collected in the Mediterranean, but this species has not been found since the original description. This species has been recorded by Shiino (1954b, 1959, 1960). According to Izawa and Choi’s (2000) direct observations of Shiino’s material: his “*C. dieuzeidei*” from *Acanthopagrus schlegeli* (Shiino 1954b) was identical with *Caligus latigenitalis*, as already pointed out by Lin et al. (1994), and his material of “*C. dieuzeidei*” from *Siganus fuscescens* (Houttuyn, 1782) (Shiino 1959) was *Caligus oviceps* Shiino, 1952 (Izawa and Choi 2000). Shiino’s (1960) specimens of “*C. dieuzeidei*” collected from two elasmobranchs were identified as an unknown congener, although the evidence was not presented. Excluding Shiino’s (1960)*C. dieuzeidei*, a total of 19 species, including the two new species described herein can be assigned to this species group. Although *C. dieuzeidei* Brian, 1932 and *C. latigenitalis* were not listed as members of the species group by Boxshall (2018), it is clear that leg 4 and the genital complex and abdomen of the female could fall within the diagnosis (see Brian 1932, Shiino 1954a, b, Izawa and Choi 2000). Unfortunately, leg 2 was neither figured nor mentioned in the text by Brian (1932) in his original description of *C. dieuzeidei*, so the configuration of the spines on leg 2 exopod cannot be confirmed.

The hosts and geographical distributions of the members of the newly recognised species group are summarized in Table 3. The host fish for the species group vary widely and include both pelagic and benthic taxa. The host specificity seems to be relatively low in *C. acanthopagri*, *C. hobsoni*, *C. ligatus*, and *C. xystercus*, but may be higher in other species. Four members of the species group most frequently utilize the family Sparidae as hosts: *C. acanthopagri*, *C. dieuzeidei*, *C. latigenitalis* and *C. xystercus*. Two species are associated with each of the following host families: Atherinidae (*C. ligatus*, *C. olsoni*), Aulostomidae (*C. ligatus*, *C. xystercus*), Pomacanthidae (*C. hobsoni*, *C. xystercus*), and Priacanthidae (*C. priacanthi*, *C. xystercus*). This is the first record of the occurrence of a species (*C. chinglonglini* sp. nov.) belonging to the species group in plankton samples. According to Venmathi Maran et al. (2016), 11 species of *Caligus* were found exclusively from plankton samples. Of these pelagic caligids, only *C. adunctus* is assigned to a species group, the *C. macarovi* group, while the remaining species have not as yet been classified into the five groups defined by Boxshall (2018).

**Table 3.** Body size, host and locality of species of the *Caligus pseudorhombi* species group.

Species	Body length (mm)	Host	Locality	References
*C. acanthopagri*	♀3.79 ♂ 5.35	*Acanthopagrus schlegeli* (Bleeker, 1854) *A. berda* (Forsskål, 1775), *Rhabdosargus holubi* (Steindachner, 1881) *Scatophagus argus* (Linnaeus, 1766), *Thryssa hamiltonii* Gray 1835,	Taiwan, south Africa	Ho and Lin 2004b
*C. bifurcus*	♀5.4	*Lateolabrax japonicus* (Cuvier, 1828)	China	Shen 1958
*C. buechlerae*	♀4.77–5.40 ♂3.58–3.85	*Tripterygion* sp.	New Zealand	Hewitt 1964
*C. chinglonglini* sp. nov.	♂4.02	–	Japan	Present study,
*C. dieuzeidei*	♀5.8 ♂6.5	*Diplodus sargus* (Linnaeus, 1758)	Mediterranean	Brian 1932, Shiino 1954b, Izawa and Choi 2000
*C. hobsoni*	♀2.78–3.45 ♂3.9	*Chromis punctipennis* (Cooper, 1863), *Hypsypops rubicundus* (Giard, 1854), *Rhacochilus toxotes* Agassiz, 1854, *Medialuna californiensis* (Steindachner, 1876)	California	Cressey 1969
*C. kajii* sp. nov.	♀4.86–6.16 ♂4.09–5.73	*Platycephalus* sp.	Japan	Present study
*C. latigenitalis*	♀3.24–4.33 ♂4.1–6.9	*Acanthopagrus schlegeli*	Japan	Izawa and Choi 2000
*C. ligatus*	♀3.20–3.35 ♂2.25–2.65	*Aulostomus chinensis* (Linnaeus, 1766), *Sargocentrus xantherythrum* (Jordan & Evermann, 1903), *Atherionomorus insularum* (Jordan & Evermann, 1903)?, *Dascyllus albisella* Gill, 1862, *Acanthrus dussumieri* Valenciennes, 1835, *Naso hexacanthus* (Bleeker, 1855)	Hawaii	Lewis 1967
*C. longirostris*	♀5 ♂6	*Pseudophycis barbatus* Gunther, 1863, *Platycephalus bassensis* Cuvier, 1829	Tasmania	Heegaard 1962
*C. musaicus*	♀3.75–5.07 ♂3.25–3.64	*Platichthys flesus* (Linnaeus, 1758)	Portugal	Cavaleiro et al. 2010
*C. nuenonnae*	♀4.27–4.82 ♂3.99–5.2	*Latris lineata* (Foster, 1801)	Tasmania	Andrews et al. 2009
*C. olsoni*	♀3.8 ♂3.8	*Leuresthes tenuis* (Ayres, 1860)	California	Pearse 1953
*C. pectinatus*	♀3.43	*Eopsetta jordani* (Lockington, 1879)	California	Shiino 1965
*C. pseudorhombi*	♀4.42 ♂3.96	*Pseudorhombus arsius* (Hamilton, 1822)	Australia	Boxshall 2018
*C. priacanthi*	♀2.9 ♂1.9	*Priacanthus hamrur* (Forsskål, 1775)	India	Pillai 1985
*C. pterois*	♀5.8 ♂4.4	*Pterois russelii* Bennett, 1831, *P. miles* (Bennett, 1828)	India	Pillai 1985
*C. similis*	♀4.95 ♂4.72	*Neophrynichthys latus* (Hutton, 1875)	New Zealand	Ho et al. 2005
*C. xystercus*	♀2.3	*Anisotremus virginicus* (Linnaeus, 1758), *Aulostomus maculatus* Valenciennes, 1841, *Calamus calamus* (Valenciennes, 1830), *C. pennatula* Guihenot, 1868, *Lutjanus apodus* (Walbaum, 1792), *Pomacanthus arcuatus* (Linnaeus, 1758), *Heteropriacanthus cruetatus* (Lacepède, 1801)	Belize	Cressey 1991

Males of 16 species belonging to the *C. pseudorhombi* group are known, including the present two new species. With the exception of *C. buechlerae*, these males can be divided into two sub-groups on the basis of the morphology of the genital complex: in one sub-group, both of the anterior and posterior knobs representing leg 5 are produced posteriorly, and leg 6 (genital operculum) is armed with 1 (or rarely 2) small setae terminally; whereas in the other sub-group only the posterior (exopodal) knob is distally produced, and leg 6 has 2 (or rarely 3) setae terminally. The first sub-group consists of *C. kajii* sp. nov., *C. musaicus*, *C. nuenonnae*, *C. pseudorhombi*, *C. priacanthi*, and *C. pterois*. The second sub-group comprises *C. acanthopagri*, *C. chinglonglini* sp. nov., *C. dieuzeidei*, *C. hobsoni*, *C. latigenitalis*, *C. ligatus*, *C. longirostris*, *C. olsoni* and *C. similis*. Members of the first sub-group are widely distributed in the Indo-Pacific and the Atlantic, whereas the second sub-group is restricted to the Pacific (Kurian 1949; Pearse 1953; Shen 1958, Heegaard 1962; Hewitt 1964; Lewis 1967; Shiino 1965; Cressey 1969; Pillai 1985; Izawa and Choi 2000; Ho and Lin 2004b; Ho et al. 2005; Andrews et al. 2009; Cavaleiro et al. 2010; Boxshall 2018; present study) (see Table 3).

Five distinct species groups within the genus *Caligus* were defined by Boxshall and El-Rashidy (2009) and Boxshall (2018), namely: *C. bonito* Wilson, 1905 (12 spp. based on Boxshall 2018), *C. confusus* Pillai, 1961 (15 spp.), *C. diaphanus* von Nordmann, 1832 (15 spp.), *C. macarovi* Gusev, 1951 (42 spp.), and *C. productus* Dana, 1852 (14 spp.). In addition, as Boxshall (2018) has already pointed out, a sixth species group, the *C. pseudorhombi* group (19 spp.) is proposed in this study. See Boxshall (2018) for the detailed definition of each species group before using the key.

## Supplementary Material

XML Treatment for Caligus chinglonglini

XML Treatment for Caligus kajii
